# KAOS: a new automated computational method for the identification of overexpressed genes

**DOI:** 10.1186/s12859-016-1188-1

**Published:** 2016-11-08

**Authors:** Angelo Nuzzo, Giovanni Carapezza, Sebastiano Di Bella, Alfredo Pulvirenti, Antonella Isacchi, Roberta Bosotti

**Affiliations:** 1 Business Unit Oncology, Nerviano Medical Sciences srl, Nerviano, MI 20014 Italy; 2 Department of Clinical and Experimental Medicine, University of Catania, Catania, 95125 Italy; 3 Department of Bioengineering, University of Applied Sciences, Vienna, 1190 Austria

**Keywords:** Gene fusion, Outlier, Gene expression, Kinase

## Abstract

**Background:**

Kinase over-expression and activation as a consequence of gene amplification or gene fusion events is a well-known mechanism of tumorigenesis. The search for novel rearrangements of kinases or other druggable genes may contribute to understanding the biology of cancerogenesis, as well as lead to the identification of new candidate targets for drug discovery. However this requires the ability to query large datasets to identify rare events occurring in very small fractions (1–3 %) of different tumor subtypes. This task is different from what is normally done by conventional tools that are able to find genes differentially expressed between two experimental conditions.

**Results:**

We propose a computational method aimed at the automatic identification of genes which are selectively over-expressed in a very small fraction of samples within a specific tissue. The method does not require a healthy counterpart or a reference sample for the analysis and can be therefore applied also to transcriptional data generated from cell lines. In our implementation the tool can use gene-expression data from microarray experiments, as well as data generated by RNASeq technologies.

**Conclusions:**

The method was implemented as a publicly available, user-friendly tool called KAOS (Kinase Automatic Outliers Search). The tool enables the automatic execution of iterative searches for the identification of extreme outliers and for the graphical visualization of the results. Filters can be applied to select the most significant outliers. The performance of the tool was evaluated using a synthetic dataset and compared to state-of-the-art tools. KAOS performs particularly well in detecting genes that are overexpressed in few samples or when an extreme outlier stands out on a high variable expression background.

To validate the method on real case studies, we used publicly available tumor cell line microarray data, and we were able to identify genes which are known to be overexpressed in specific samples, as well as novel ones.

**Electronic supplementary material:**

The online version of this article (doi:10.1186/s12859-016-1188-1) contains supplementary material, which is available to authorized users.

## Background

Kinases are key enzymes that regulate several critical cellular processes related to cell proliferation. Their over-expression and activation, as a consequence of gene amplification or gene fusion events, is a well-known mechanism of tumorigenesis and their protein products represent ideal targets for the development of novel anti-cancer drugs [1].

With the increasing availability of new potent and selective kinase inhibitors, the ability to identify fusion proteins in different cancer types has the potential to influence diagnostic and therapeutic decisions, allowing the identification of individuals who would benefit from specific kinase inhibition, according to the paradigm of personalized medicine for cancer treatment.

The identification of rare events of kinase over-expression in tumor subtypes can be used as a hallmark of underlying genomic rearrangements, leading to the expression of the target genes in a tissue where they are normally not significantly expressed. An example is the expression on NTRK1 in colorectal cancer (CRC). NTRK1 is normally not expressed in colon but it becomes overexpressed once the region encoding its catalytic kinase domain is fused with the 5′portion of TPM3 gene, as the consequence of an intra-chromosomal inversion, resulting in the TPM3-NTRK1 fusion gene [2]. Similarly, ALK is normally not expressed in lung, while it has been found overexpressed and activated in non small cell lung cancer samples (NSCLC) when fused to EML4, following a chromosomal translocation [3]. Other examples include rearrangements involving ROS1, FGFRs, RET, MET or EGFR ([4–9]).

The search for novel rearrangements of kinases or other druggable genes may therefore contribute to the understanding of the biology of cancerogenesis, as well as lead to new candidate targets for drug discovery.

Recently, screening of cancer samples from The Cancer Genome Atlas (TCGA) [10] showed that kinase rearrangements are rare events that can be detected in few tumor samples across a specific tumor type [11]. Therefore there is a specific need for new computational methods that can allow the specific detection of rare recurring rearrangements events.

Cancer cell lines can be used as a model to identify new kinase oncogenes and to study their sensitivity to drugs. However, this requires the ability to query large datasets to identify rare events occurring in a very small fraction (1–3 %) of different tumor subtypes. This task is different from what is normally done using established computational methods that are able to find differentially expressed genes between two experimental conditions, or through strategies focused on the search for outlier genes in a specific tumor type.

Several methods have been reported to detect genes with an outlier expression profile using different algorithms for cancer outlier profile analysis. COPA (Cancer Outlier Profile Analysis) [12] searches for pairs of genes (potential gene fusions) with a large number of outliers in tumour samples and few or none in normal samples. The Gene Tissue Index (GTI) algorithm [13] is based on a statistical method derived from economics, which determines whether there is a significant increase in the expression of a specific gene in a sub-group of tumours compared to the group of normal samples. For each group, it calculates a statistical index that represents the proportion between the number of outlier samples and the total number of samples. The indexes are then compared to determine the differences between the two groups. Both methods require transcriptional data from both tumour and normal tissue counterpart and cannot therefore be applied in the cases where normal counterpart is not available or in the profiling of cancer cell lines.

Other approaches, such as ZODET [14] or the method proposed by Kothary and colleagues [15], search for abnormalities in the gene expression profile of an individual compared to a reference population. While GTI and COPA search sub-populations of samples for outlier expression levels of a gene, in this case sub-groups of genes (or a single gene) are analyzed in a single sample and the algorithm searches for the gene(s) showing the highest expression value both in absolute terms and with respect to a reference population. In particular, ZODET is based on Z-score outlier detection and it has been implemented for use on Illumina whole genome microarray data. Also in this case, a reference control is required. All the methods described so far use microarray data as input. On the contrary, Kothary’s method has been implemented to receive in input RNASeq data. It requires gene expression levels expressed as RPKM, a gene expression measure that might not be ideal especially when used for inter-sample analysis [16]. The method searches for outlier kinases by comparing gene absolute expression level (RPKM) versus its differential expression with respect to the median expression value of the same gene across samples. It quantifies the statistical significance of each outlier by means of Mahalanobis distance [17]. One limitation of the method is that it has not been implemented as a standalone tool, but it requires R [18] for statistical analysis and an external tool for visualization (GraphPad Prism) [19].

Other bioinformatics methods have been developed that do not rely on gene expression data as an indirect readout of gene rearrangements, but search for unbalanced 5′/3′ gene expression or try to locate gene fusions genomic breakpoints. For example, Cancer Gene Census [20] compares expression levels of all the proximal versus distal exons for each exon-exon junction, in order to predict the existence of transcriptional breakpoints. Other methods try to predict fusion genes at the genomic level by checking genes in their transition regions or analyzing Copy Number Variations (CNVs) [21]. Finally, several tools, such as TopHat, FusionFinder or FusionMap are based on RNASeq data and detect gene fusion candidates by considering the discordant read pairs aligning to two different genes, as reviewed in Carrara et al. [22]. The main limitation of such tools resides on the high number of false positives contaminating the results produced [23].

In this paper, we propose a new computational method for the automatic identification of genes selectively over-expressed in a small fraction of tumour samples within a specific tumor tissue type, while taking into account its expression level also in other tumor subtypes. Indeed, although certain kinase fusions only rarely occur in a specific tumour type, they have been shown to recur when multiple tissue types are considered [11].

The method does not require a healthy counterpart or a reference sample for the analysis and can therefore be applied to transcriptional data generated from clinical samples as well as from cell lines. In our implementation, the tool is independent from the platform used to generated gene expression data and can be used with data obtained using microarrays as well as Next Generation Sequencing (NGS) RNASeq technologies.

The method has been implemented as a publicly available, user-friendly tool called KAOS (Kinase Automatic Outliers Search, http://www.nervianoms.com/en/component/phocadownload/category/2-kaos.html?download=2:kaos). The tool enables the automatic execution of the iterative search for outliers and visualization of the results using a graphical interface with filters for the selection of the most significant outliers. As case study, for validation purposes, we used publicly available microarrays data performed on 917 cell lines belonging to 24 different tumor types form the Cancer Cell Line Encyclopedia (CCLE [24]) and showed that the tool is able to detect genes which are known to be overexpressed in certain tissue samples as well as to identify novel ones.

## Methods

**Fig. 1:**
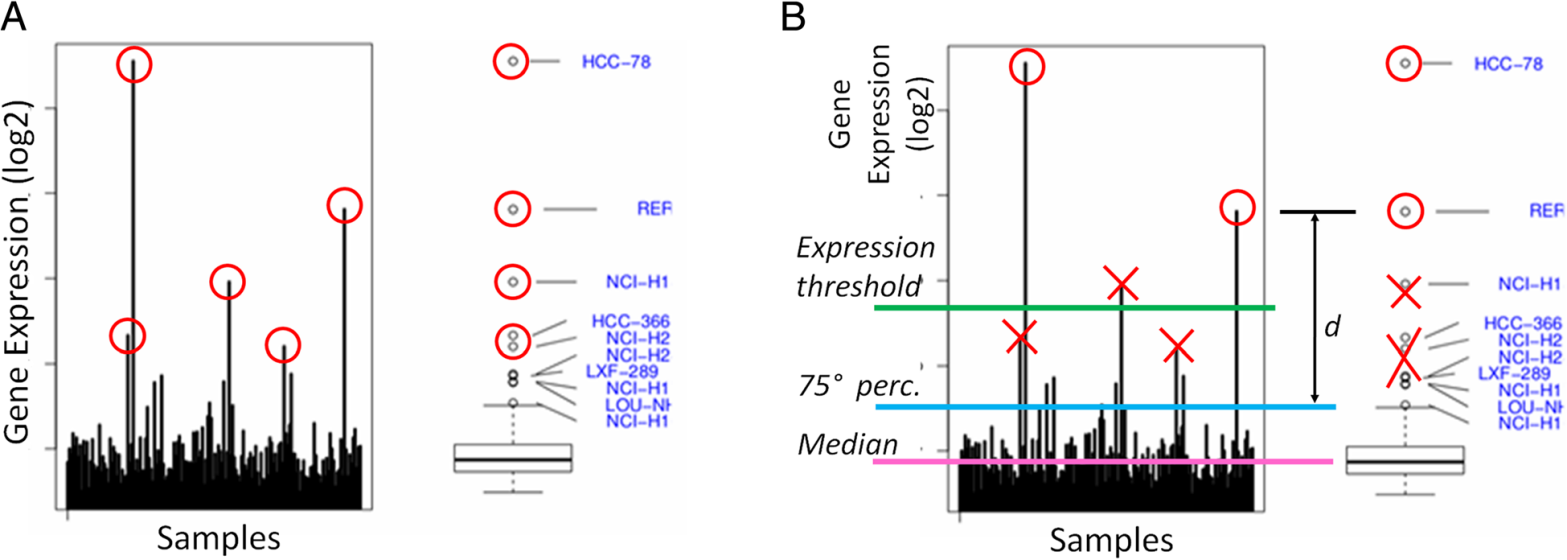
Outlier detection. Outlier detection method is reported. **a** Statistical detection: for each kinase, gene expression level in all the analysed samples belonging to a specific tumor type is reported as an histogram (*left panel*) and as boxplot (*right panel*). “Rare events” (a kinase over-expressed in one or a few cell lines and low/not expressed in the others) are identified by mean of the Grubb test and reported as a red circle. **b** Prioritization and filtering: the most relevant outlier kinases are selected applying specific filter criteria (minimal expression threshold; maximum median level of expression over the tumor type; minimum distance from the 75th percentile of the tissue-specific distribution; proportion of the number of outliers with respect to the whole dataset of outlier occurrences). Samples that do not consistently pass the imposed filters are removed (reported in the figure as red crosses)
iii) Our objective is to identify samples with an extreme distribution for the examined gene in the tumoral tissue under consideration, even when the background expression level is relatively high. Therefore, specific filtering criteria are applied in order to prioritize the more robust outliers. From the set of computed outliers, the KAOS algorithm selects the gene expression values fulfilling the following criteria (see Fig. 1b):
  the expression value is above a minimal expression threshold
  the gene has a minimum median (med) level of expression in the tissue-specific distribution
  the value has a minimum distance (d) from the 75th percentile of the tissue-specific distribution
iv) The remaining outliers are then ranked using the “non-dominated sorting” ranking algorithm, commonly used in multi-objective optimization field [26], which allows to select the best solution on the basis of two or more metrics.
  The ranking algorithm iteratively checks if each outlier i dominates over any other outlier j in the set, such that: if a) all the distances of i are greater than or equal to the corresponding distances of j, and b) at least one of distances of i is strictly greater than the corresponding distance of j, then i is assigned rank 1. Once all the outliers identified with rank 1 are discarded from the outliers set, the same comparison is performed to assign rank 2. The same iteration is repeated to assign all greater ranks until the set is empty. Figure 2 shows a bi-dimensional example on a 2-metrics computation.

ᅟ The tool implements the following strategy:
  i) pre-processing of the dataset to provide tissue of origin annotation;
  ii) identification of statistical outliers for each tissue-specific distribution;
  iii) application of a chain of filtering criteria to isolate tissue-specific outliers;
  iv) ranking of the outliers in order to eliminate potential false positives;
  v) provision of a graphical summary of the most relevant outliers and related gene expression distributions.
i) The first step consists in the annotation of the tumor samples under investigation with information on the tissue of origin (in the current version of the tool, this is a manual annotation pre-processing step, which can be achieved using external annotation tool or custom software scripts).
ii) Once a group of cell lines or tumoral samples is grouped according to the tissue they belong to, a distribution of the gene expression values is computed and plotted together with a box-and-whiskers plot. We used the R statistical environment, which implements the Grubb test for outliers [25]. The result of the test includes all the values statistically considered outliers, independently of the absolute gene expression value. These lead to a high number of outliers since, in principle, the most extreme values of any distribution might be considered outliers (Fig. 1a).

**Fig. 2:**
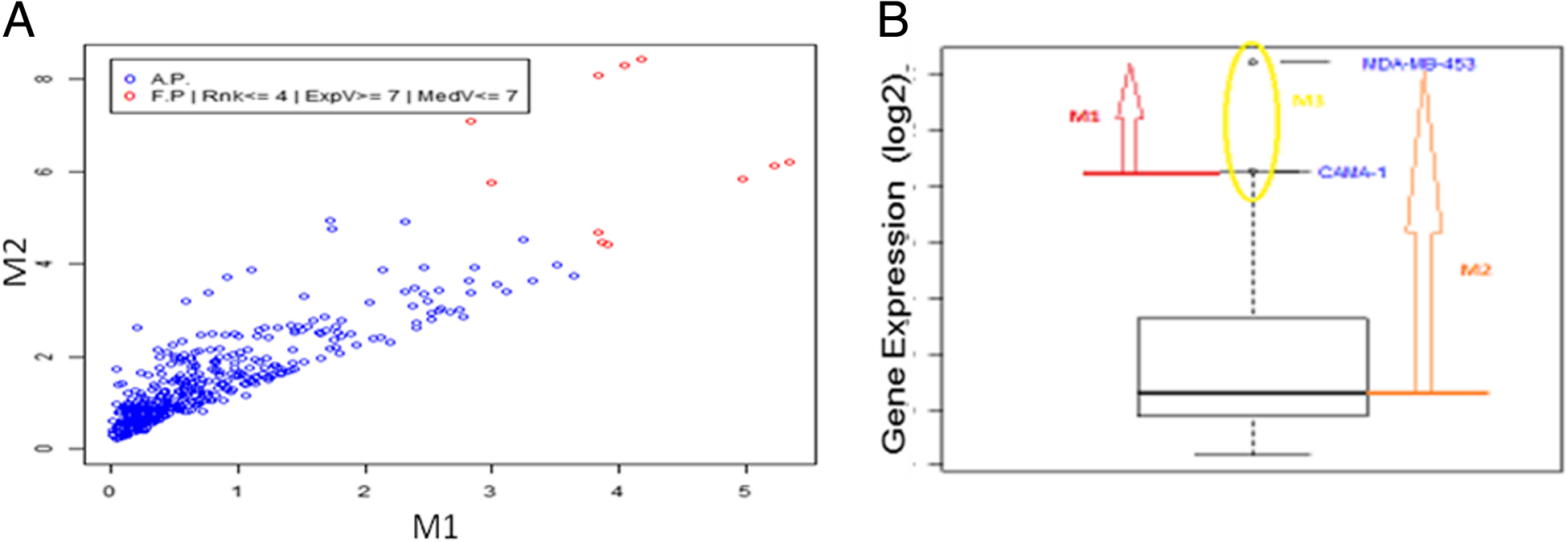
The ranking algorithm. **a** 2-d plot of the two measured distance: M1 is the distance from the upper wisker; M2 is the distance from the median. The “best” outliers lie on the top right corner of the graph, that corresponding to a major distance from both upper wisker and median, and are reported as red dots. **b** The metrics used for ranking are reported: M1 (*red arrow*) is the distance from the upper wisker; M2 (*orange arrow*) is the distance from the median; M3 (*yellow circle*) is the number of samples in which the gene has an outlier expression value
  In order to obtain the optimal settings for the detection of the extreme outliers, we used three metrics:
  the distances of the gene expression level from the 75th and the 50th percentile of the tissue-specific distribution
  the proportion of the number of outliers with respect to the whole dataset of outlier occurrences for the given gene (a proportion that should be kept <5 %).

To allow a user-friendly interaction in parameter setting and visualizing of the results, as well as to enable an interactive use of the search strategies, we provided the method with a graphical interface.

The algorithm is meant to be applied to any gene expression dataset. We developed and tested it on microarrays data, but it can be easily applied to RNA-seq data. Since we wanted the tool to be independent of the dataset under investigation, we designed it to interact with a database schema which should be implemented by the user in a standard RDBMS, as described in the Results and Discussion section.

## Results and discussion

### Method implementation

Discovering candidate rearrangement in a panel of tumour cell lines on the basis of their gene over-expression could be seen as a multidimensional problem, thus claiming for a systematic and automated approach. While a manual visual inspection of the expression pattern of a specific gene in a cell line is rather trivial, it is more complicated to extend the same analysis on the genome scale and on a high number of tumoral samples. This is especially true when searching for a rare event, such as the detection of the occurrence of an outlier gene expression only in few samples among a tumoral tissue type.

Here we present a computational tool, that we called KAOS (Kinases Automated Outlier Search), for the identification of rare events of samples with an outlier kinase expression. The KAOS algorithm was implemented using the R statistical environment [18]. In particular, we used the R function “boxplot-with-outlier-label” [27] to calculate the statistics and the boxplots and the function fastNonDominatedSorting of the “nsga2R” package [28] to compute the rank of each outlier.

The algorithm has then been embedded in a software tool with a graphical user interface, developed using the Java programming language [29] and a data interface to a MySQL database. The software assumes MySQL, Java and R installed on the computer. The tool is then provided as an executable file to automatically create the database and populate it with an example artificial dataset (along with the tool, for solely testing purposes) and a user guide with the instructions on how to run the application and how to prepare the input files. The tool has been tested on different platforms and can be used on Linux, Windows and Mac OS. The tool can be used on different types of gene expression data, as long as they are stored in the database using the format provided in the example schema.

**Fig. 3:**
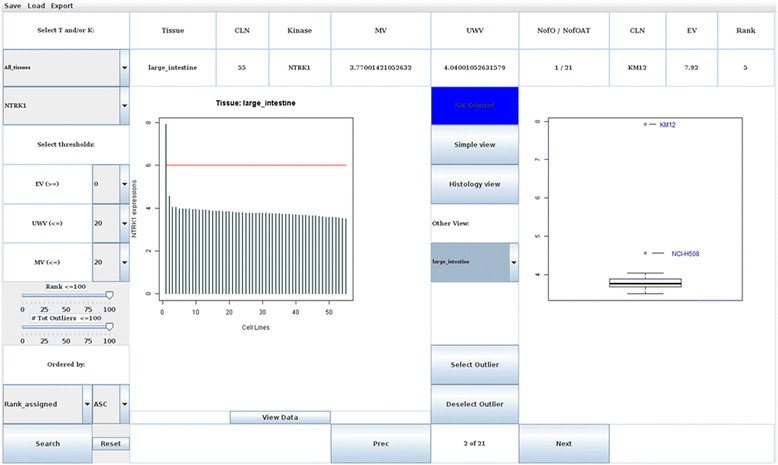
Graphical User Interface. KAOS graphical user interface, developed in Java, is reported. The interface allows to visualize both the information on the detected outliers (*top panel*) and graphically represent the results (*central panel*) at the same time. The interface allows to customize query parameters and to filter the results (*left panel*)

The graphical user interface of the tool is shown in Fig. 3. On the left-side are reported the filters that can be customized by the user for the selection of the outliers, including specification of the tumour tissue type and of the gene name of interest. For the outlier selection, a variety of statistical filter thresholds can be customized, such as gene expression level, median value and upper whisker. The user can also set the maximum threshold value for the rank and for the total number of outliers for the gene of interest in all the other tumoral tissues, allowing the identification of tumor specific, as well as general outliers. An additional function allows selecting the order in which results are sorted (by mean of gene expression values, rank, tissue or gene name). On the top left corner of the interface a menu provides the commands to save and reload the selected search criteria.

Expression level of the gene of interest in the selected tissue is shown in the left panel, while the detected outliers are shown in the box plot on the right. Command buttons allow the visualization of the expression profile of a gene in each single tumoral tissue type one by one or all together at the same time. Additional information related to each outlier is reported at the top.

### Performance evaluation on simulated data

By making use of simulated data, we compared KAOS performance with published outlier detection methods such as GTI [13], ZODET [14] and an adapted version of the method published in [15].

To test the method presented by Kothari et al. in [15], which was developed to be used on RPKM gene expression data only, we had to slightly modify it. For this purpose, we computed three metrics: i) the absolute expression within the sample, ii) the differential expression (defined as the ratio of the absolute expression and median expressions of the gene within the compendium) and iii) the Mahalanobis distance of a point x (defined using the above absolute and differential expressions) to the mean μ in each sample. We then performed a consensus analysis by claiming a gene “outlier within a sample” when it has a value greater than the upper whisker of the boxplot for all the three metrics. For each gene we then computed the number of samples in which it is recognised as outlier and we ranked the genes according to that value.

In order to perform a comparison with ZODET, we followed the same approach the authors used to compare their method with GTI. More precisely, for each gene we counted the number of samples in which it is marked as outlier and then ranked the genes according to this value.

Finally, in order to test GTI, we used the R script provided by the authors and ranked genes accordingly.

Following the simulation proposed in [13] and [14], we first generated an artificial dataset with 1000 genes having an equal number of cancer and normal samples (30 in each class). The expression values of the genes were drawn from a normal distribution having mean 7 and standard deviation 1. Such values reflect the Affymetrix microarrays data analysis standard practice of considering 6–7 as a minimal expression value, as well as the typical average found in TCGA [10] and CCLE [24] datasets. The genes assumed to be differentially expressed, named True Positive (TP), were generated by adding a constant *m* to their expression in the *k* samples which have been marked as outliers’ samples. The TP rate is 5 % (i.e. 50 genes). In order to find the simulated false positives (FP), in each simulated experiment we ranked the genes according to their score and considered as predicted outliers only the top *t* (*t* ranging from 10 to 50). Within such a computed list, we then calculated the correctly predicted TP genes and FP genes, the number of False Negative (FN) and the number of True Negative (TN). We could therefore compute the average Precision, Recall and F-Measure by running 50 simulations. We analysed the performances of KAOS, GTI, ZODET and the Kothari et al. method varying *k* from 10 to 1 and *t* from 10 to 50. Since KAOS does not need case/control data we tested it on the 30 cancer cases only.

Table 1 gives the measures obtained for *k* = 1 in all the compared tools. The results clearly show that KAOS outperforms the other tested methods in terms of Precision/Recall when the top 10 and 20 outliers are considered. In such a case KAOS seems to be the most robust method. On the other hand, when a higher threshold is applied, ZODET outperforms the other methods. Tables 2 and 3 give the measures for *k* = 5 and *k* = 10. In these cases GTI has the best performances in terms of Precision/Recall.

**Table 1:** Tools comparison on simulated data for k = 1

	k = 1, T = 10			k = 1, T = 20			k = 1, T = 50		
	Precision	Recall	F-measure	Precision	Recall	F-measure	Precision	Recall	F-measure
KAOS	**0.348**	**0.348**	**0.348**	**0.267**	**0.267**	**0.267**	0.162	0.162	0.162
Zodet	0.232	0.232	0.232	0.243	0.243	0.243	**0.220**	**0.220**	**0.220**
GTI	0.182	0.182	0.182	0.175	0.175	0.175	0.146	0.146	0.146
Khotary et al.	-	0.038	-	0.114	0.032	0.050	0.121	0.012	0.022

The comparison of Kaos performances is based on 50 simulations on a synthetic dataset made of 1000 genes expression values for 30 cases and 30 cancer test samples. The expression values were drawn from a normal distribuion with mean 7 and standard deviation 1, where *k* samples which have been marked as outliers’ samples (see Methods section for further details) and T is the top T number of outlier genes found. The table shows average Precision, Recall and F-Measure for k =1 and t ranging from 10 to 50

In bold are reported the values obtained by the best performing tool in the different conditions

**Table 2:** Tools comparison on silmulated data for k = 5

	k = 5, T = 10			k = 5, T = 20			k = 5, T = 50		
	Precision	Recall	F-measure	Precision	Recall	F-measure	Precision	Recall	F-measure
KAOS	0.526	0.526	0.526	0.374	0.374	0.374	0.244	0.244	0.244
Zodet	0.828	0.828	0.828	0.699	0.699	0.699	0.516	0.516	0.516
GTI	**0.862**	**0.862**	**0.862**	**0.773**	**0.773**	**0.773**	**0.548**	**0.548**	**0.548**
Khotary et al.	0.454	0.246	0.319	0.450	0.124	0.194	-	0.041	-

The table shows the same simulation results as Table 1 when k = 5

In bold are reported the values obtained by the best performing tool in the different conditions

**Table 3:** Tools comparison (k = 10)

	k = 10, T = 10			k = 10, T = 20			k = 10, T = 50		
	Precision	Recall	F-measure	Precision	Recall	F-measure	Precision	Recall	F-measure
KAOS	0.268	0.268	0.268	0.188	0.188	0.188	0.109	0.109	0.109
Zodet	0.986	0.986	0.986	0.948	0.948	0.948	0.765	0.765	0.765
GTI	**0.998**	**0.998**	**0.998**	**0.984**	**0.984**	**0.984**	**0.802**	**0.802**	**0.802**
Khotary et al.	0.837	0.776	0.805	0.754	0.389	0.513	0.767	0.151	0.252

The table shows the same simulation results as Table 1 when k = 10

In bold are reported the values obtained by the best performing tool in the different conditions

The obtained result confirms the value of KAOS in detecting the most extreme outliers, since the algorithm was designed and optimized with the aim of searching for very rare rearrangements and for extreme outliers in a high variability expression context. That is indeed what resembles most likely real cases, when gene rearrangements are expected to affect 1–3 % only of the investigated samples. The simulation for *k* = 1, indeed, represents the condition of a rare case, while the simulation of a high variability expression context is simulated by placing the constant *m* equal to 2. When the number of outlier samples increases (ie. *k* = 5, 10), other tools show better performance, as they have been designed for such broader search purposes.

### Method application on experimental data

The aim of the tool is not to perform comparisons across different data sets or platforms but rather to support users with the analysis of their dataset, that can be normalized according to the technology used and to user preferences.

To validate the tool on a real dataset, we tested the algorithm on about 500 kinase genes from Cancer Cell Line Encyclopedia (CCLE) gene expression dataset [24]. In this dataset 917 cell lines, belonging to 24 different tumor types, were profiled by microarrays and probe set intensities were calculated using the Robust Multi-array Average (RMA) and normalized by the quantiles method [30]. In this way, per each gene, outlier identification was performed within the same dataset and the same platform.

**Fig. 4:**
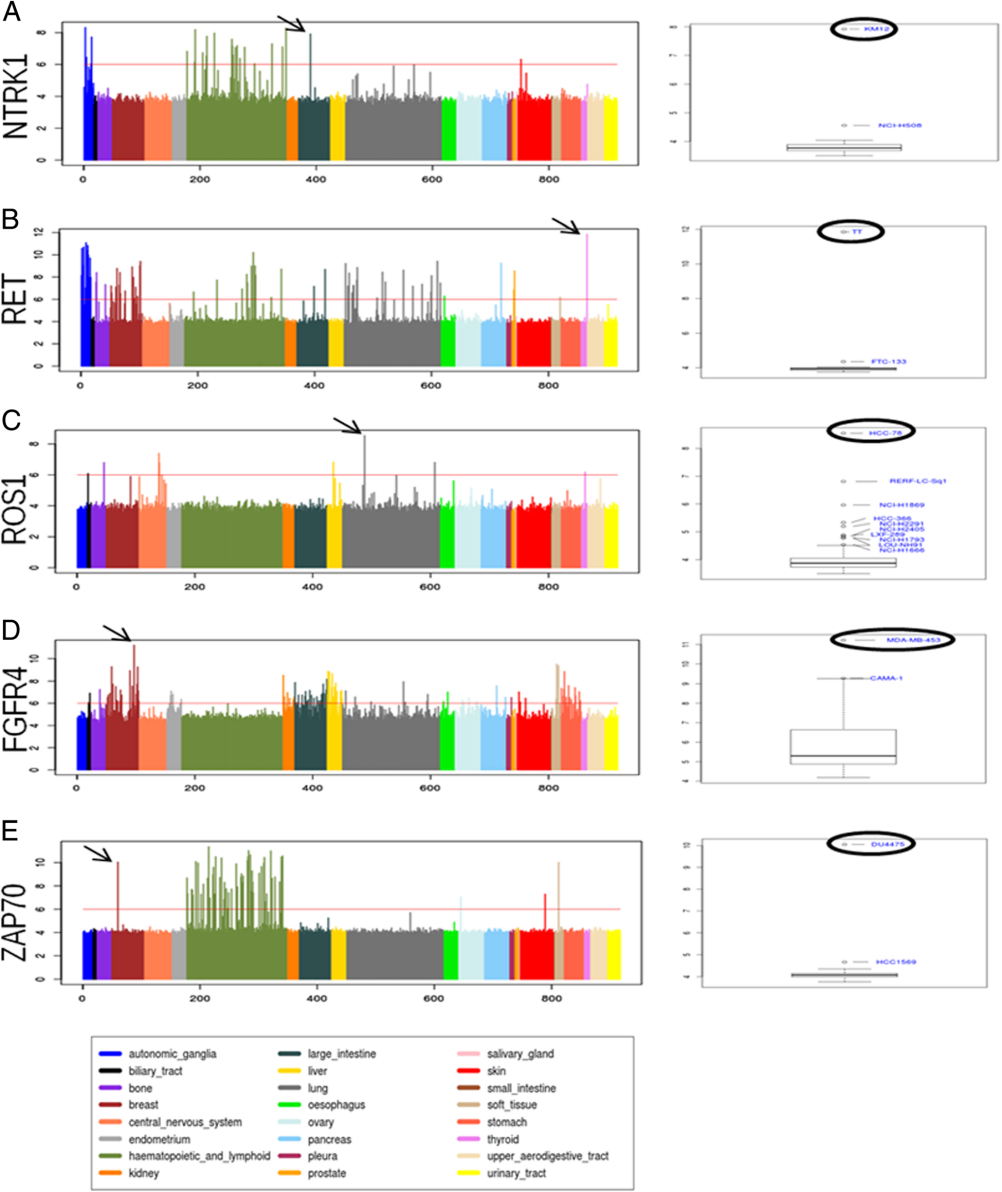
Identification of known and new overexpressed kinases. Figure 1. Left panel shows gene expression level of a selected kinase in 917 cancer cell lines belonging to 24 different tumor types (CCLE data) as histogram. Tumor types are reported in different colors. The boxplot of the tissue-specific distribution of the kinase is reported in the right panel. Outlier samples are reported as black circl. **a** NTRK1 is generally expressed in hematopoietic and lymphoid and autonomic ganglia. No expression is observed in large intestine (colon), apart in KM12 colorectal cancer cell line, highlited as outlier in this tissue; **b** RET tyrosine kinase is generally expressed in tissues such as autonomic ganglia, haematopoietic tissues, but no expression is observed in thyroid tumors. In this tissue a dramatic expression of RET can be detected in TT papillary tumor cell line only, assigned as outlier by the tool; **c** ROS1 tyrosine kinase is typically poorly expressed apart in colon where HCC-78 lung cancer cell line stands out as a clear outlier; **d** FGFR4 is highly expressed in few breast cancer cell lines, among those MDA-MB-453 breast cancer cell line appear as highly overexpressed. **e** ZAP-70 tyrosine kinase can be observed in haematopoietic and lymphoid tissues only. No expression in breast cancer cell lines can be appreciated, with the exception of a significant overexpression of the gene in DU4475 breast cancer cell line

The method was able to correctly identify several kinases known to be overexpressed in specific cell lines among a tumour tissue type. Indeed, NTRK1 was correctly identified as highly expressed in KM12 colorectal cancer cell line (Fig. 4a). NTRK1 is a tyrosine kinase typically not expressed in colorectal cancer tissue, however it become expressed and activated as consequence of a genomic rearrangement involving the C-terminal kinase catalytic domain of NTRK1 that is fused with the oligomerization N-terminal domain of TPM3, an ubiquitously expressed protein. We had previously reported the identification and characterized of the TMP3-NTRK1 rearrangement in KM12 colorectal cancer cell line and demonstrated that over-expression of NTRK1 in this setting is the driver event of tumorigenesis and renders tumors sensitive to NTRK1 kinase inhibitors in preclinical models [3]. Similarly, we could detect an overexpression of the ROS1 tyrosine kinase in HCC-78 lung cancer cell line only, within lung tumor cancer cells (Fig. 4b). ROS1 is indeed overexpressed as a consequence of a genomic translocation that leads to the expression of a chimeric FIG-ROS gene [31]. Also, the system allowed the identification of a significant overexpression of RET in the TT cell line, among thyroid papillary tumour cell lines (Fig. 4c). In this case RET overexpression and activation is not the result of a rearrangement, but it is a consequence of a mutation event that leads to the expression and activation of the kinase [32]. Moreover, FGFR4 overexpression was observed in MDA-MB-453 breast cancer cell line, among breast cancer cells. Also in this case the anomalous activation of the kinase is a consequence of the presence of a Y367C oncogenic mutation [33].

The analysis also allowed highlighting unexpected kinases over-expression. This is the case of ZAP-70, a tyrosine kinase expressed in T lymphocytes, in which it plays a role in initiation and amplification of T-cell receptor signalling. ZAP-70 is selectively expressed in tissues of lymphoid origin and is not typically observed in solid tumours [34]. Indeed, in Fig. 4e the expected homogeneous expression distribution of ZAP-70 in lymphoid tissue can be observed but, correctly, no outliers are detected by KAOS in the hematopoietic and lymphoid tissues, as no extreme outlier value stands out over the high variability context. On the other hand, using KAOS, we could detect an anomalous expression of the gene in a single breast cancer cell line, the DU4475 (Fig. 4e), as an extreme outlier out of 56 breast cancer analysed samples. ZAP-70 expression was further investigated in the DU4475 cell line by western blot, using a specific antibody (sc-1526) against the C-terminal domain of the protein, confirming the high level of ZAP-70 expression also at protein level (see Additional file 1). No expression of ZAP-70 could be appreciated in MCF7 breast cancer cell line, used as control.

The functional relevance of ZAP-70 overexpression in DU4475 breast cancer cell line is currently under investigation.

## Conclusions

Discovering candidate rearrangements in a panel of cancer cell lines on the basis of their anomalous gene expression in few samples only could be seen as a multidimensional problem, thus claiming for a systematic and automated approach. While a manual visual inspection of the expression pattern of a specific gene in a single sample is rather trivial, it is more complicated to extend the same analysis on the genome scale and on a high number of samples. This is especially true when searching for a rare event, like the detection of the occurrence of the expression of a gene in few samples (outliers) only.

To this aim we developed KAOS, a user-friendly tool for the rapid and efficient detection of rare events of kinase overexpression in specific tissues. The tool uses gene expression data either from microarrays or RNASeq technologies.

The performance of the tool was evaluated with a synthetic dataset and compared to the state-of-the-art tools. KAOS performed particularly well in detecting extreme outliers that stands out on a high variable expression background.

We provided an example of application using gene expression data for the detection of kinase over-expression, but the analysis could be easily extended to other gene families.

The tool represents a concrete example of how the increasing overwhelming availability of genomic knowledge bases, which are still growing over time, can be exploited for new target discovery.

## Additional file

Additional file 1: Protein expression of ZAP70 in DU4475 breast cancer cell line. Characterization by Western Blot analysis of ZAP-70 protein. Total cell lysated were subjected to Western Blot analysis using anti-ZAP70 (sc-1526) goat polyclonal antibody raised against a peptide mapping at the C-terminus of ZAP-70. 1) DU4475 (20 ng); 2) MCF7 (20 ng); 3) HisGST-ZAP70 recombinant protein (15 ng), positive control. (PNG 68 kb)

## Abbreviations

CCLE: Cancer cell line encyclopedia
CNVs: Copy number variations
COPA: Cancer outlier profile analysis
CRC: Colorectal cancer
FN: False negative
FP: False positive
GTI: Gene tissue index
KAOS: Kinase automatic outliers search
NGS: Next generation sequencing
NSCLC: Non small cell lung cancer
RDBMS: Relational database management system
RMA: Robust multi-array average
RPKM: Reads per kilobase per million
TCGA: The cancer genome atlas
TN: True negative
TP: True positive
ZODET: Z-score Outlier DETection
