# Supplementary figures and images for: KAOS: a new automated computational method for the identification of overexpressed genes
Source: BMC Bioinformatics. 2016 Nov 8;17(Suppl 12):5–14. doi: 10.1186/s12859-016-1188-1 (PMC5123341; doi:10.1186/s12859-016-1188-1)

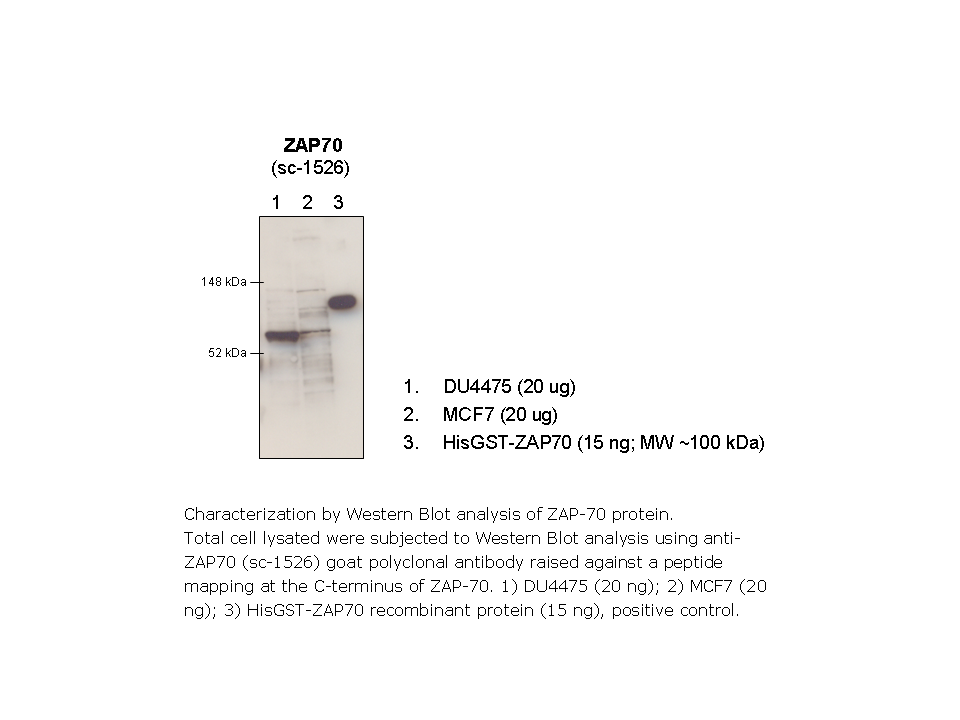

Supplement: Additional file 1: — Protein expression of ZAP70 in DU4475 breast cancer cell line. Characterization by Western Blot analysis of ZAP-70 protein. Total cell lysated were subjected to Western Blot analysis using anti-ZAP70 (sc-1526) goat polyclonal antibody raised against a peptide mapping at the C-terminus of ZAP-70. 1) DU4475 (20 ng); 2) MCF7 (20 ng); 3) HisGST-ZAP70 recombinant protein (15 ng), positive control. (PNG 68 kb) [file 12859_2016_1188_MOESM1_ESM.png]
